# Correction: Loss-of-function mutation of NSD2 is associated with abnormal placentation accompanied by fetal growth retardation in mice

**DOI:** 10.1371/journal.pone.0334633

**Published:** 2025-10-14

**Authors:** Eriko Ohnishi, Shiori Kinoshita, Kazuhiko Nakabayashi, Kenichiro Hata, Tomoko Kawai

S1 Table is uploaded incorrectly. Please see the correct S1 Table here.

## Supporting information

S1 Table Statistical-test p-values in the comparisons of body and placental weights between wild-type and heterozygous/homozygous individuals. (XLSX)
