# Supplementary figures and images for: Loss-of-function mutation of NSD2 is associated with abnormal placentation accompanied by fetal growth retardation in mice
Source: PLoS One. 2025 Jul 21;20(7):e0328243. doi: 10.1371/journal.pone.0328243 (PMC12279110; doi:10.1371/journal.pone.0328243)

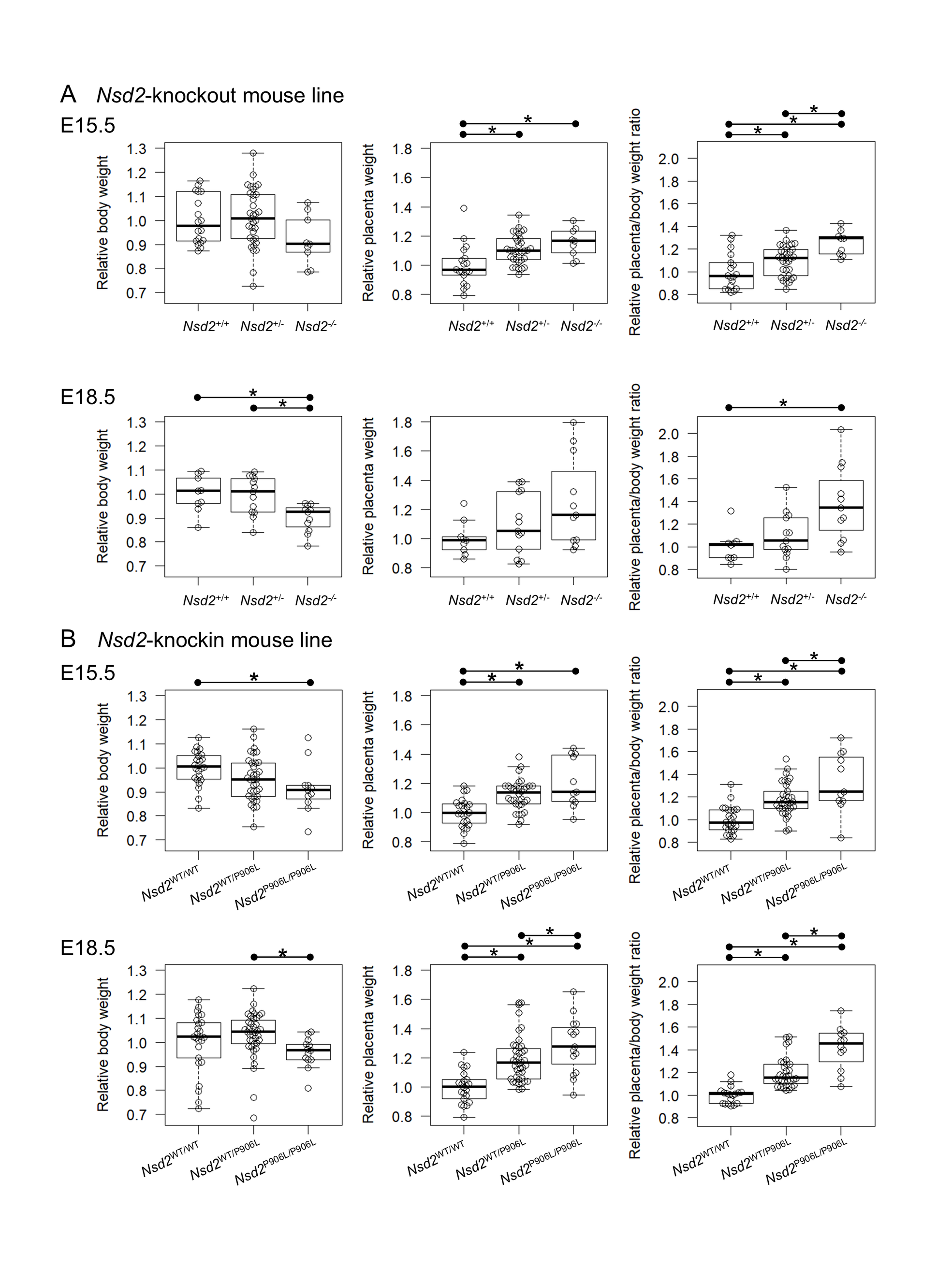

Supplement: S1 Table — (TIF) [file pone.0328243.s001.tif]

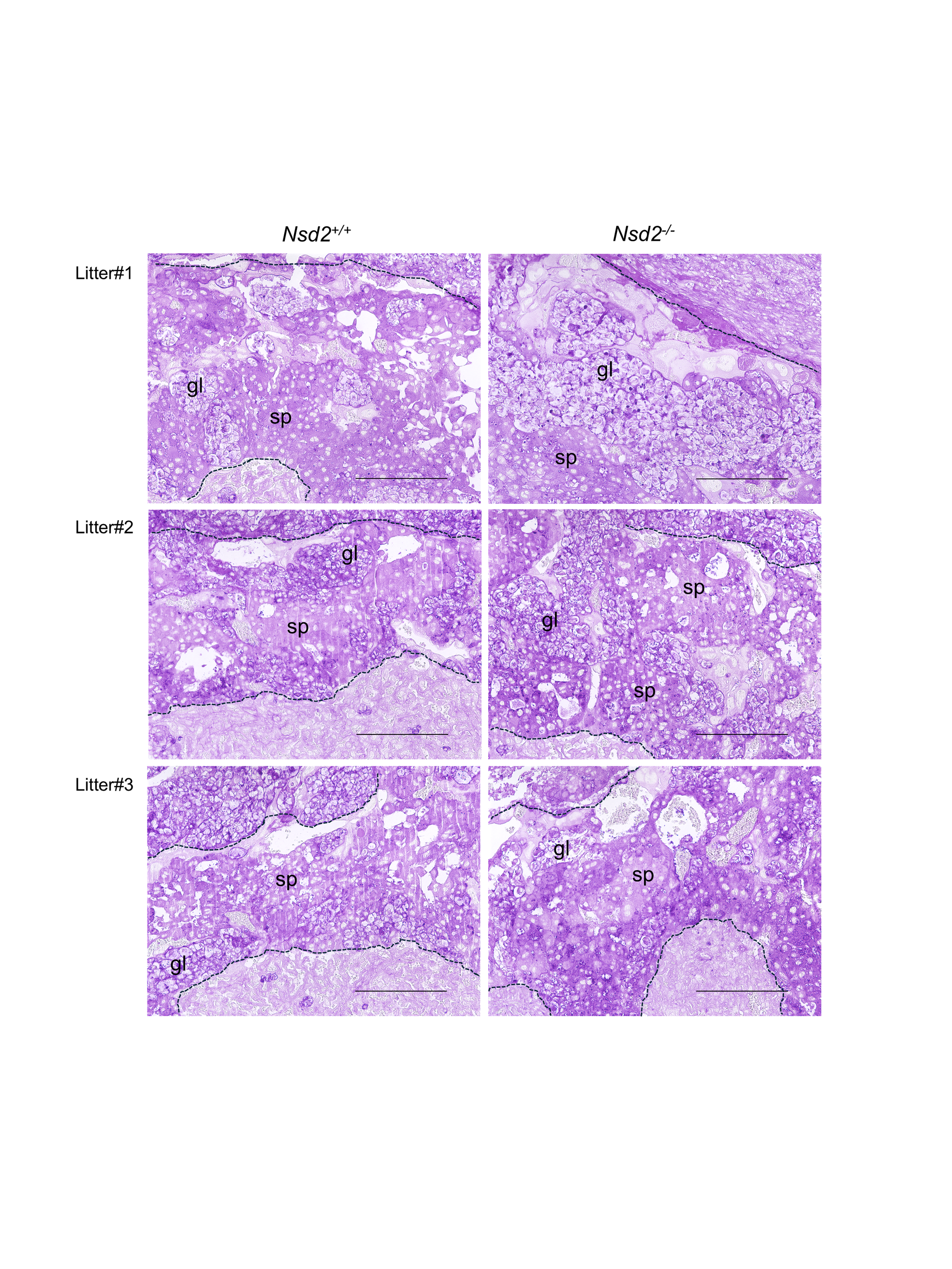

Supplement: S1 Fig — PAS-stained sections of Nsd2-/- and Nsd2WT/WT littermates are shown. The top figure shows a section of the same tissue as shown in Figure 2. Dashed lines indicate the boundaries of the three layers. gl, glycogen trophoblast cells (foamy appearance); sp, spongiotrophoblast cells. Sale bar = 200 μm. (TIF) [file pone.0328243.s002.tif]

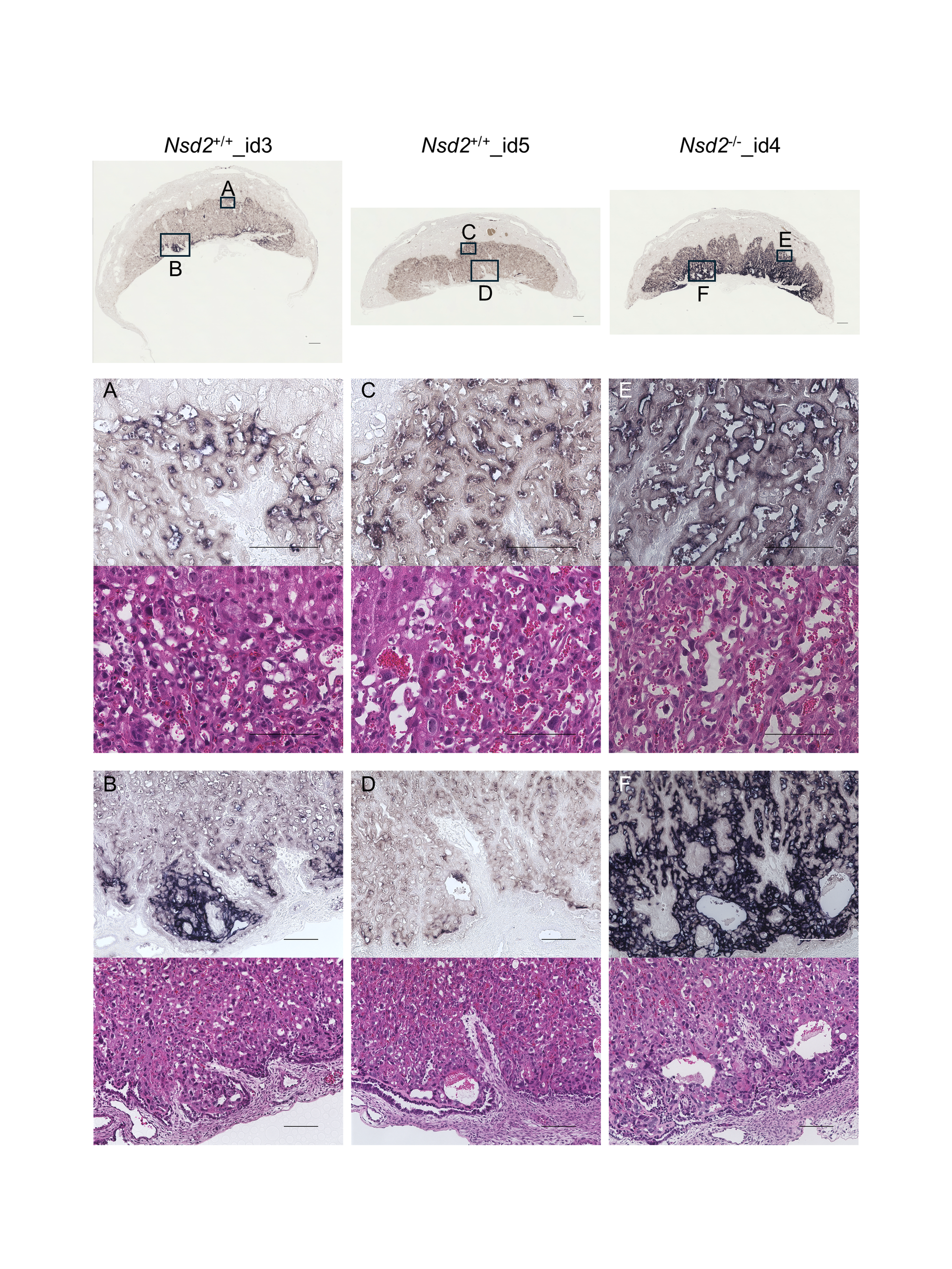

Supplement: S2 Fig — One Nsd2-/- (id4) and two Nsd2+/+ (id3 and id5) littermate placentas were subjected to alkaline phosphatase staining. Boxes labeled with corresponding alphabets represent the enlarged areas shown in A-F. HE-stained serial sections are shown below each magnified image. Sale bar = 300 μm (top) and 100 μm (magnified images). (TIF) [file pone.0328243.s003.tif]

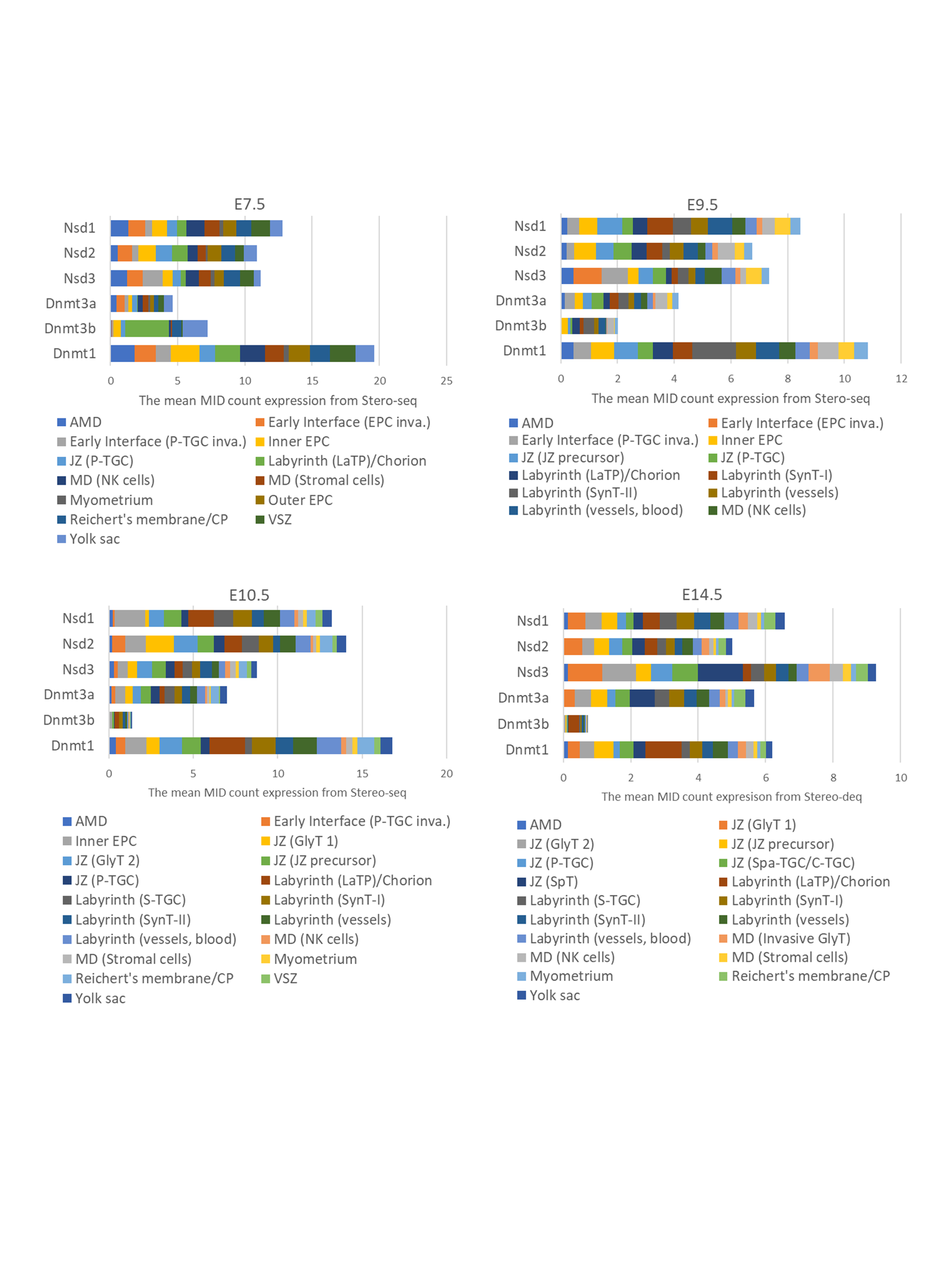

Supplement: S3 Fig — Bar graphs show the cell type composition of gene expression using publicly available single-cell based spatial transcriptome data spanning from E7.5 to E14.5. [14]. (TIF) [file pone.0328243.s004.tif]
