# Supplementary material for: Loss-of-function mutation of NSD2 is associated with abnormal placentation accompanied by fetal growth retardation in mice
Source: PLoS One. 2025 Jul 21;20(7):e0328243. doi: 10.1371/journal.pone.0328243 (PMC12279110; doi:10.1371/journal.pone.0328243)
Supplement: Attachment — Submitted filename: Response_letter_250605.docx [file pone.0328243.s007.docx]

PONE-D-24-42707

Title: **Response − Loss-of-function mutation of NSD2 is associated with abnormal placentation accompanied by fetal growth retardation in mice**

Dear Editor,

Thank you for giving us the opportunity to revise our manuscript. Your valuable feedback helped us to improve our manuscript. We have thoroughly reviewed all the comments and have made appropriate revisions to address these points in the manuscript.

**Editor’s comments**:

The authors have carefully addressed most of the criticisms of the reviewers and the editor. However, statistical analysis requires further clarification. The underlying issue appears to be that pairwise ratios (that reduce the variation in the control “values” to zero) appear to have been used but are not warranted by the experimental design. However, the method used is not clearly described so some ambiguity remains. The individual experimental and individual control observations/values do not appear to be methodologically paired in any empirically meaningful way. Without this built into the experimental design, paired values cannot be used to determine ratios. Instead, ratios must be determined based on the control values as a group and the variability of the control values (the ratios of individual control values to the mean of all control values) must be included in the statistical analyses. One way to do this is to calculate the ratios of the experimental and the control values to the mean of the control values. The experimental and control ratios can then be used to test null hypotheses in a statistically valid manner.

Description of the statistical analysis does not unambiguously state whether paired or non-paired values were used. Doing so will help clarify which statistical analyses are appropriate.

**Response**: Thank you for pointing these critical issues to our attention. We have clearly described the method for determination of ratios in the statistical analyses. We calculated the ratios of the experimental and the control values to the mean of the control values in this revised manuscript. Subsequently, we implemented a non-paired Wilcoxon rank sum test between the groups. Pairwise comparisons were performed between the three genotypes. We described these methods in lines 104 to 107 in “Mouse phenotyping and statistical analysis” in a revised manuscript.
